# A New Quantum Video Processing Algorithm Based on the NEQR Technique

**DOI:** 10.3390/e28020168

**Published:** 2026-02-01

**Authors:** Adrian Prodan, Alexandru-Gabriel Tudorache, Vasile Manta

**Affiliations:** Department of Computer Science and Engineering, “Gheorghe Asachi” Technical University of Iasi, D. Mangeron Street nr. 27A, 700050 Iasi, Romania; adrian.prodan@academic.tuiasi.ro (A.P.); vasile-ion.manta@academic.tuiasi.ro (V.M.)

**Keywords:** quantum image processing (QIP), quantum circuit, quantum algorithm, segmentation, NEQR, Qiskit

## Abstract

The main goal of this paper is to present a new way of processing a video file using a combination of multiple quantum methods. The design is built upon the novel enhanced quantum representation technique, NEQR, which is then expanded using ideas such as image segmentation, implemented with the help of one or multiple comparators, binarization and cycle shift. This approach allows us to process all frames in parallel according to the desired parameters—one or more thresholds. A demonstration circuit for the proposed design, using a couple of frames, that sums together all the concepts is implemented using the Python programming language and Qiskit open-source framework, made available by IBM. The circuits are analyzed in the experimental section, using the Simulator component and configured using the noise properties of real devices, where we present different relevant metrics obtained by processing the simulation results.

## 1. Introduction

The evolution of the quantum universe has seen another rebirth in the last years, both in terms of research ideas and quantum processors developed by big corporations. There is a considerable amount of money being raised toward quantum efforts, more than $44.5 billion worldwide according to [1]. These numbers are justified by their potentially promising applications: artificial intelligence (AI), cybersecurity, energy, drug development, image processing, random number generation, finance optimization, traffic flow improvement, weather forecasting, and others (see [2] for details).

### 1.1. Related Work

Over the years, the image processing field has been a heavily researched topic among professors and scholars, with papers carefully investigating various mapping techniques over quantum technology, both from a technical and an experimental point of view. This gave birth to what we refer to now as quantum image processing (QIP). Depending on the type of image, we mention ideas that build on the representation of gray scale images, using a vector of angles for color representation (called FRQI—flexible representation of quantum images, see [3]); extensions of these notions, with multiple phase-parameters for different color channels (for the RGB representation), have also been presented by some authors (an improved FRQI model called FRQCI [4], and another improved model that transitions from angles to rotation matrices—IFRQI [5]).

Other concepts seem to be easier to apply regarding their implementation using different programming languages and their quantum packages; one such example is the NEQR technique (novel enhanced quantum representation [6]), used for grayscale images. Unlike FRQI, the authors of NEQR use the basis state of a sequence of qubits for their gray level component. The authors of paper [7] show an experimental comparison of the NEQR scheme with other image representation techniques, some based on the Quantum Fourier Transform (QFT), with an emphasis on the quality of the image and efficiency; the performance of these representations is analyzed in terms of gate errors, using a local GPU (graphics processing unit) and a QPU (quantum processing unit). More schemes have been proposed for various types of images (or color models), and different review papers offer a broader overview of their impact, implementation, and potential use-cases, with their general advantages and disadvantages [8,9]. There have also been papers that aim to reach image segmentation goals, which are based on merging diverse concepts together (such as image representation techniques and comparator blocks that implement custom threshold levels) with a reduced quantum cost [10]. Paper [11] presents an algorithm and its corresponding circuit for operating on a grayscale sequence of images, using the background difference method; the authors use multiple basic blocks for image processing operations to achieve a quantum circuit, and evaluate it with the help of a quantum simulator.

In terms of ideas regarding the varied quantum blocks that can be applied in the circuit design (for QIP applications), we first mention a concept that focuses on performing the general comparison operation between values represented using n bits [12]; this quantum comparator, applicable on multiple bits, is implemented via a single ancilla qubit. Another paper that targets a reversible layout is one that works in stages, is performance optimized, and based on prefix trees [13]; according to the authors of the mentioned design, this idea results in superior performance when compared to blocks that use the prefix-based comparator. A different approach analyzes the cost of quantum blocks and proposes a new way of minimizing the cost of comparator circuits with the help of Clifford+T gates, thus improving the integration of error correction codes [14]. The authors build a robust design set apart by a lower cost, with properties that make it resilient to noise from external sources.

Binarization is an extremely important operation in the image processing field, which basically transforms every pixel of the image to black or white; it can be applied in more complex circuits for segmentation purposes, if we want to either locate an area of the image, or perhaps find an object with certain properties. Proposals for circuit architectures that implement efficient binarization operations, based on quantum comparators, have been discussed in different papers, with the simulation results used to check the correctness of the ideas [15]. We also mention a design that proposes fault-tolerant comparators in the quantum area with the aim of binarization [16]—in this mentioned paper, the authors minimize the noise impact on their circuits by employing an optimized number of T gates. There have been approaches over the years that rely more on the physics component to analyze their potential viability as image processing techniques—these ideas are built on quantum dots technology (or semiconductor nanocrystals); image operations such as smoothing and edge detection have been studied by some researchers using the cellular neural network model [17]. Concepts such as Quantum-dot Cellular Automata (QCA) have been investigated for designing quantum circuits with image processing applications that present good performance in terms of error and efficiency [18]—the idea of a 5-input fault-tolerant majority gate is key to obtaining superior performance, allowing for advancements in the nano technology area. Papers that explore other quantum concepts for video encryption algorithms have been published; relying on an improved logistic map, controlled-XOR operations, as well as frame watermarking and corresponding circuits, have been theoretically analyzed and simulated, having the common goal of proposing secure and efficient schemes for video applications [19,20]. Other articles that further investigate the quantum video processing topic have also been recently published [21,22,23,24,25].

### 1.2. QIP Applications

It is very important for the readers to consider that the results of QIP research papers are not just theoretical; applications of the QIP field are extremely vast and will have a profound effect on a wide array of areas, effectively shaping the future of our planet. Medical use-cases and their impact on the healthcare sector are being researched: quantum computing for drug discovery, quantum sensing for analyzing neurological diseases, quantum key distribution (QKD) for transferring sensitive medical data, and so on [26,27]. The intersection and viability of quantum technology with the blockchain field are topics that have been also studied by some authors, with properties such as transparency and resistance to attacks, secured by cryptographic algorithms, being highly regarded on the internet of today’s society [28]. Recommendations of system designs that unify the blockchain field with requests for quantum processors accessible in the cloud have been examined; integrated into smart contracts, they could increase public trust in the quantum domain and play a significant educational role as a quantum advocate [29]. Other commercial applications of quantum computing in fields such as cybersecurity, materials, banking, and manufacturing have also been studied [30]. Using artificial intelligence methods (such as ideas for combining neural networks and quantum computing into a hybrid processing model) is becoming more prevalent, with authors analyzing various architectures, both from a software and hardware point of view, that can tackle such an important challenge [31,32].

In this manuscript, we first focus on processing an image using quantum properties for segmentation purposes, then propose, analyze and run experiments for a way to extend and customize a quantum circuit for working with a small sequence of images.

Our paper is structured as follows: Section 2 presents the general concepts that represent the fundamentals of our proposed design; Section 3 shows the implementation details and the circuits for a simple example; Section 4 discusses the performance results in terms of time and error using a quantum simulator, with the experiments taking place on the local GPU; Section 5 highlights the main conclusions of the analyzed work and a potential development road.

## 2. Materials and Methods

### 2.1. General Architecture

Our main objective is to find a way to process a video file (multiple frames) that is more efficient if implemented using a quantum circuit than in a classical manner. By investigating and utilizing the discovered quantum properties, such as entanglement and superposition, we managed to design a quantum circuit for this purpose, by combining specific ideas and processing blocks.

The system that we propose and analyze starts with one of the possible quantum representations of images; there are multiple choices available here, but for ease of implementation, control of position and pixel color (gray level), as well as reputation, gained by being selected by multiple researchers over the years, we decided to choose the novel enhanced quantum representation technique (NEQR). In NEQR, the color component of images represents a gray level (value) in a given range.

We can then process a simple video file (composed of multiple frames) by implementing a certain number of image operations after the NEQR representations (in terms of circuit gates).

In order to apply a segmentation idea when building a quantum circuit, we can first try to find one or two gray levels, which act as thresholds (knowing that the pixels of our searched object contain values only in the range given by those limits); then, we can either apply two comparator blocks (one to first detect pixels with values above a certain level, and the second for further selecting those with levels below a given threshold), or directly apply a block that keeps the desired pixels, if possible.

**Figure 1:**
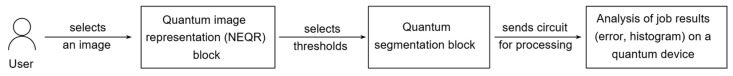
Workflow for a simple image, using the NEQR representation.

The general workflow for a single grayscale image is presented in Figure 1, which shows the steps that a user should take to obtain the corresponding quantum circuit.

**Figure 2:**
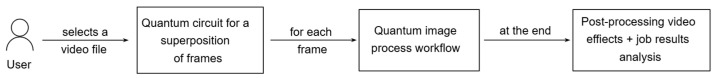
Workflow for a sequence of images, obtained by extending the previous ideas.

This general idea is then adapted for a simple sequence, as shown in Figure 2. The workflow for such a video file further develops the schematic in Figure 1, but the first logical layer is the creation of a superposition of frames; each one is then processed (contains a circuit component) according to the mentioned ideas regarding a single image. There is also an optional final layer that includes different effects (or final processing blocks) for the whole video, or more exactly, for the desired pixels of one or more of the indicated images.

The last block in Figure 2 encapsulates post-processing ideas (video effects) and the analysis of the probability histogram, which can be used to verify the correctness of our circuit, and/or further interpret and use the actual qubit measurements in a more complex project. The post-processing concepts are not the objective of this paper and can be seen as further development ideas; generally speaking, depending on how we want to approach this final computing block in regards to the measurement process, these concepts can be implemented in two ways: either in a quantum manner (similar to the previous blocks in the process workflow), or classically—if we refer to more complex operations, where the quantum circuit no longer brings any significant performance improvements. In the second case, we might decide it is more computationally efficient to implement the rest of our operations in an ordinary processing environment, after measuring the qubit states (by running the simulation for a given number of program runs, known as shots), thus creating a hybrid computing scheme.

### 2.2. Building Blocks

**Figure 3:**
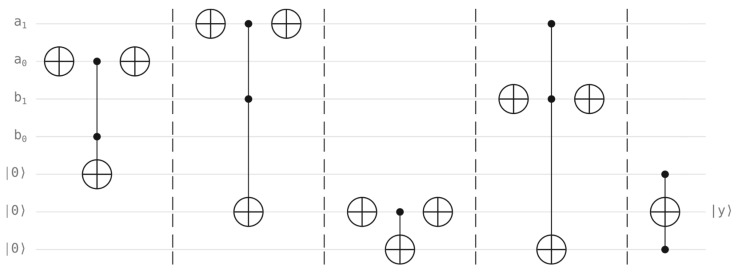
Comparator circuit for two qubit registers. The circuit compares register $\left|a_{1}a_{0}\right\rangle$ with register $\left|b_{1}b_{0}\right\rangle$ to find whether the first value is greater than or equal to the second. Each register is made of two qubits, and the result is stored using the second auxiliary qubit (marked with y notation).

One of the building blocks implemented in our workflow is the comparator design (and its circuit), described in detail in paper [10]. Figure 3 presents this circuit, which compares two values, each requiring two qubits for their representation; the circuit can be obtained by either configuring the Qiskit package in a local development environment [33,34] or directly by using the IBM Composer website [35].

By examining the comparator in Figure 3, we can identify three groups of qubits:

- the first comparison value, stored in the first qubit register $\left|a_{1}a_{0}\right\rangle$;
- the second comparison value, stored in the second qubit register $\left|b_{1}b_{0}\right\rangle$;
- three auxiliary qubits (the last three qubits) in the lower part of the circuit.

The output is stored in the second of the last three auxiliary qubits and referred to as |y⟩ on the right side. The convention for the output qubit is given in Equation (1):

(1) $$y=\left\{\begin{array}{c} 0,\;if\;a\geq b\\ 1,\;if\;a<b \end{array}\right.$$

**Figure 4:**
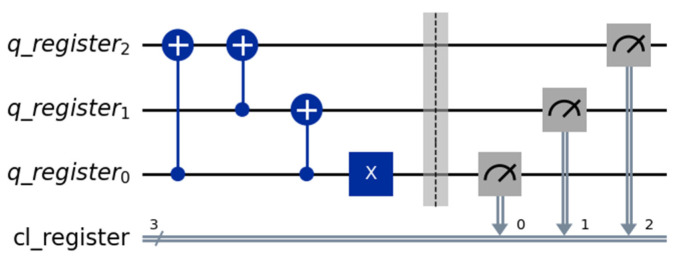
Simple cycle shift “+” operation.

**Figure 5:**
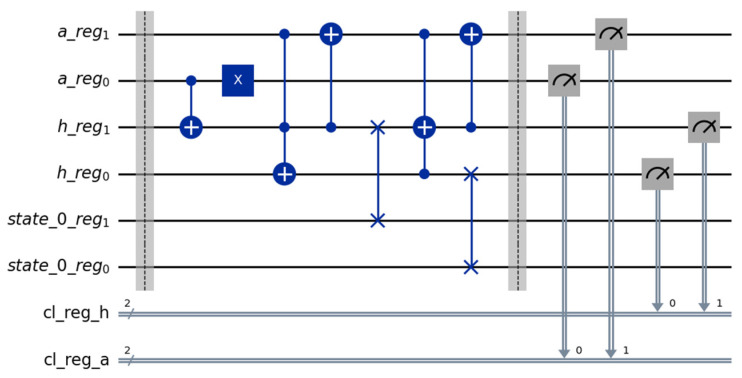
Circuit implementation of the optimized cycle shift operation “+” (for increasing the frame index).

Regarding the quantum video processing component, there are multiple approaches available; one of them is based on a relatively trivial implementation of the “+” cycle shift operation, shown in Figure 4, with more complex ideas presented in paper [11] (Figure 4 and Figure 5 are obtained from the mentioned paper). The “+” operation allows us to increase the index of the current frame from our video sequence, while “−“ calculates the index of the previous frame.

**Figure 6:**
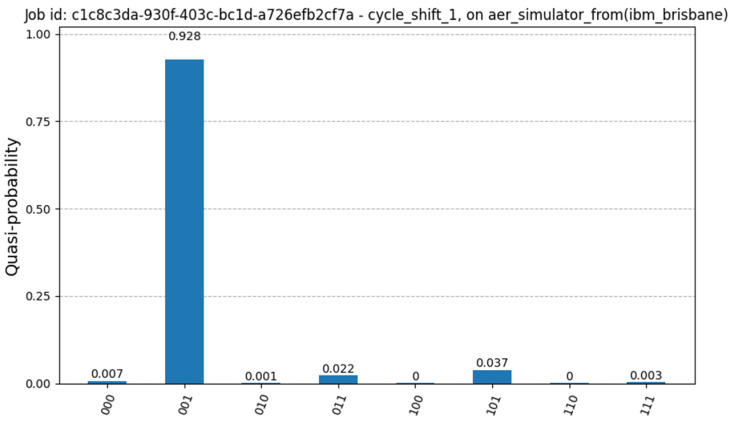
Simulation results for the simple cycle shift “+” operation.

Figure 4 presents the circuit configured for a 3-qubit register, with the initial state set to |000⟩. Figure 6 illustrates the measurement results, using the simulator component in Qiskit, based on the error configuration from a real quantum device (“ibm_brisbane”), which shows the next state correctly identified as |001⟩.

**Figure 7:**
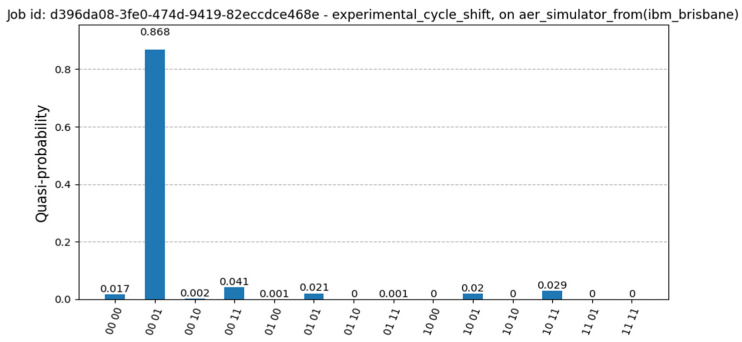
Measurement results of the optimized cycle shift from Figure 5. The current state is set to |00⟩, and the next state is correctly identified as |01⟩.

Figure 5 shows the circuit based on the idea from paper [11], implemented in Python using Qiskit, where the authors describe a method for a more efficient cycle shift. Here, the initial state is |00⟩, with simple measurements showing the highest probability of the next state of |01⟩ in Figure 7, using the previous configuration for the simulation.

## 3. Implementation Details

The circuits and their implementations are presented in this section, for a simple example, using a sequence of two frames (we selected a small number of frames, with figures of small sizes for demonstration purposes; this reduces the number of qubits for the size component, the selection of the image, and enhances the clarity of the circuit).

The operations implemented in Qiskit are described as follows:

- The representation of quantum images (the NEQR technique);
- The comparator circuit, which we can configure to use as a segmentation tool for the detection of pixel positions within certain gray level ranges;
- A selection operation (based on the CNOT gate or the cycle-shift operation), in order to progress in the video and keep detecting the relevant sections for each frame of an image.

We now present the core elements of each implementation item. The circuit for each operation (or set of operations) is presented below, followed by the circuit for all the operations put together.

### 3.1. NEQR Circuit for a Test Image

We first generated four test images, each with gray values ranging from 0 to 255, each having four pixels (sized 2 × 2); these values are as follows:

- the first image:      $\left[\begin{array}{cc} 0 & 75\\ 200 & 255 \end{array}\right]$;
- the second image: $\left[\begin{array}{cc} 0 & 100\\ 170 & 255 \end{array}\right]$;
- the third image:    $\left[\begin{array}{cc} 0 & 125\\ 140 & 255 \end{array}\right]$;
- the fourth image:  $\left[\begin{array}{cc} 0 & 150\\ 110 & 255 \end{array}\right]$.

**Figure 8:**
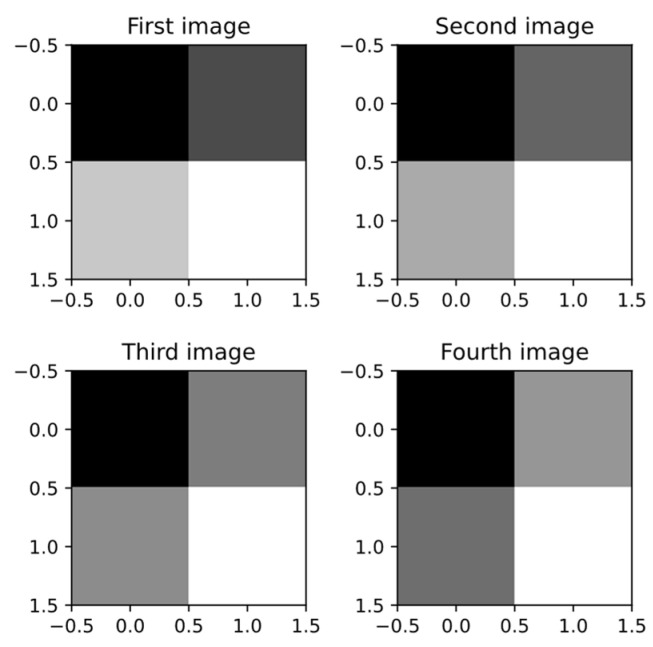
The four test images, each sized 2 × 2.

The test images, with the gray level values set as previously mentioned (the four gray level frames), are visually represented in Figure 8; they represent the images of our test sequence.

**Figure 9:**
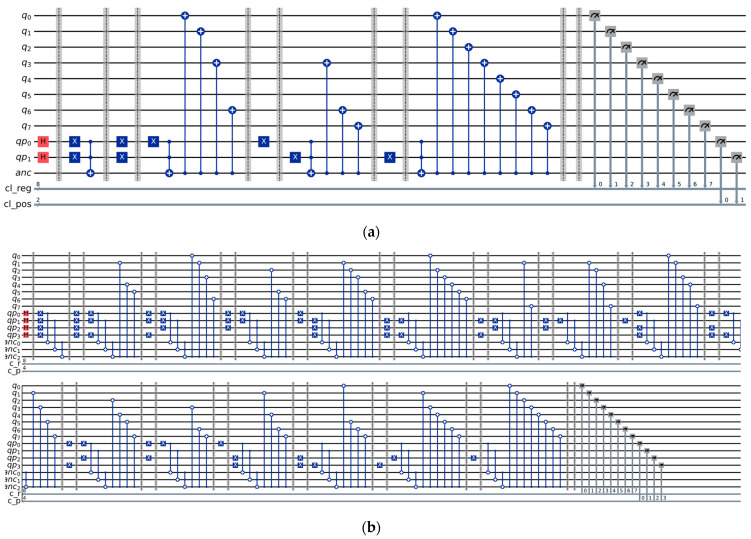
(**a**) NEQR representation of the first test image; (**b**) Experimental NEQR circuit for a 4 × 4 test image.

The NEQR technique, selected to represent the first test image, can be consulted in Figure 9a; similar circuits are used to represent all the images.

The circuit in Figure 9a contains 11 qubits, grouped in the following qubit registers:

- $q_{7}-q_{0}$: an 8 qubit register for the gray value of the current pixel;
- $qp_{1}-qp_{0}$: a 2 qubit register for the position of the pixel (one qubit for the row index and one for the column);
- anc: an ancilla qubit, that is set to the |1⟩ state when the position qubits are in the desired state; it is then used to set the gray level of the corresponding value qubits, with the help of CNOT gates.

The measurement results of this circuit generate four “spikes” that correspond to the actual gray values of the pixels (we did not attach the histogram since we have $2^{10}=1024$ values on the X axis—we are measuring 10 qubits: 8 for the gray levels and 2 for the position). The probability of the other states, although considerably diminished, is still present, and will start making an impact on the circuit as we increase the number of qubits; the simulation took place on the simulation component (Aer) using the noise model of IBM Strasbourg, a real quantum machine.

In order to show how such a circuit can be scaled, the NEQR representation for a 4 × 4 image (with randomly selected values) is presented in Figure 9b.

The additional qubits for the circuit in Figure 9b (when compared to the circuit in Figure 9a) are required for the position and auxiliary components: 4 position qubits instead of 2, along with 2 more ancilla qubits.

### 3.2. The Comparator Circuit

One way to build the comparator circuit would be to extend one of the existing ideas to represent the whole 8 qubit register for the current pixel value, then use the same idea for the threshold value. After that, if our segmentation purpose would require a given range, we would re-append the comparator block, but this time configured with a different threshold value. This would indicate the pixels (the potential region or regions of the image) that fall between certain limits.

In order to simplify the implementation, reduce the number of qubits, and better align with the purpose of our project, we decided to opt for a custom segmentation approach; this only indicates pixel positions with gray level values that respect a condition in relation to the threshold value—not the entire value, but most significant two bits. This way, we make use of a comparator designed for two registers of two qubits each.

**Figure 10:**
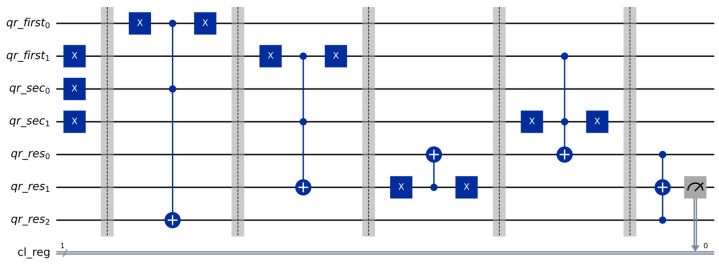
Comparator circuit for 2 qubit registers.

Figure 10 presents the implementation of the comparator circuit (described in paper [10] and explained in Section 2) to indicate if the first value is greater than or equal to the second.

**Figure 11:**
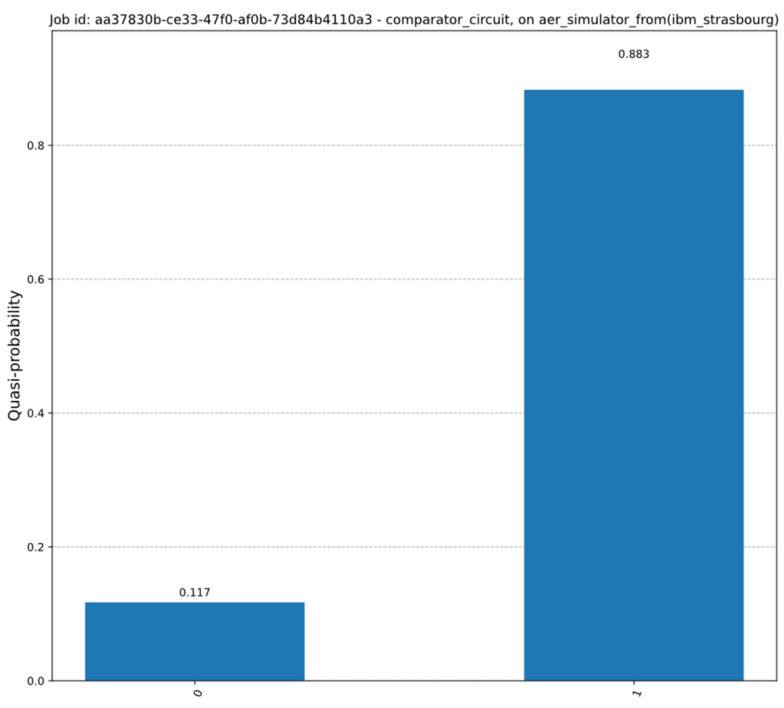
Simulation results for the comparator circuit.

The measurement results (the circuit being tested on the local simulator) can be observed in Figure 11. For this test circuit, the first register is set to state |01⟩ and the second is set to |11⟩. The probability histogram indicates a much higher probability for the measured value of 1, which respects the convention, since the second value is greater than the first.

**Figure 12:**
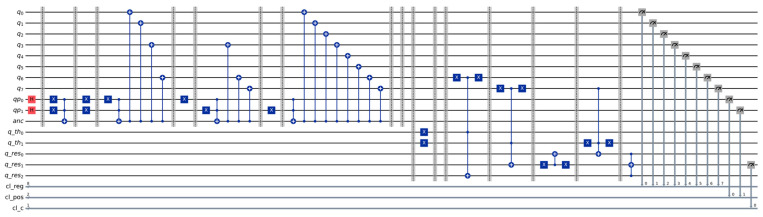
Circuit for NEQR representation of the first image and comparator circuit.

Figure 12 shows the implementation of the NEQR representation combined with the comparator circuit, where we set the comparison values as follows: the first register is set to the qubit state of the two most significant bits of the gray value register, while the second qubit is set to |11⟩. The purpose of this design is to detect pixel values that are higher than or equal to 192 (its binary value is $11000000_{2}$).

For the circuit illustrated in Figure 12, we used 16 qubits, as follows:

- The upper part of the circuit is the NEQR representation and requires 11 qubits, as previously described;
- The two threshold qubits in the |q_th⟩ register, $\left|q\_th_{1}q\_th_{0}\right\rangle$, are set to |11⟩, while the rest of the configuration for the comparator is not modified;
- As for the interpretation, the two most significant qubits of the image ($\left|q_{7}q_{6}\right\rangle$) play the role of the first register, compared to the second one (|q_th⟩).

### 3.3. The Complete Circuit

**Figure 13:**
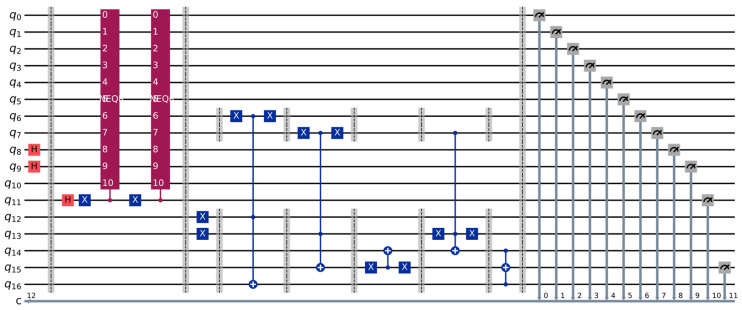
The demonstration circuit, with the NEQR representation of the first and second images as the two NEQR blocks, a qubit for the image selection and the comparator circuit.

The cycle shift concept is merged with the previous ideas with the purpose of designing a demonstration circuit; bearing in mind the limitations of the simulator—having a reasonable number of qubits—we chose to use the NEQR representation for two images (for testing purposes), together with the comparator circuit; one extra qubit was also added to our design, which can be used to select the current image (it acts as a CNOT gate, connected to the NEQR blocks). This circuit can be seen in Figure 13.

For this circuit, the following quantum registers are required, summing 17 qubits in total:

- The first 10 qubits ($q_{10}-q_{0}$) are used for the NEQR representation of the images, as already analyzed; for this test, the $q_{9}$ and $q_{8}$ qubits indicate the position and are set in superposition only once, before the first barrier (this operation is removed from the NEQR blocks);
- Qubit $q_{11}$ acts as a selector/simple cycle shift, since this circuit is designed for two images; it is first set in superposition (using a Hadamard gate), and acts overall as the control qubit of a CNOT gate, selecting the current representation of one of the two images depending on its state;
- Qubits $q_{12}$ and $q_{13}$ are used to indicate the threshold value, and they are set to the |11⟩ state; we use the same ideas of the most two significant qubits as previously shown in Figure 12;
- Qubits $q_{14},\;q_{15}$ and $q_{16}$ are the auxiliary qubits from the comparator circuit, with $q_{15}$ holding the result.

In terms of measurement, we use the following convention for our classical register (starting with the most significant bit): comparator result (1 bit), image selector (1 bit), position indices (2 bits) and image gray level value (8 bits). More details on the actual values that we are searching for and how to interpret them using the simulation results (including the measured counts for each state) are given in the experimental section of this paper.

## 4. Simulation Results

### 4.1. System Setup

For this section, we focus on analyzing relevant metrics of the experimental results. Having developed our architecture using known blocks and ideas such as the NEQR technique, we find it crucial to further explore parameters of such a circuit in an experimental scenario.

Building a complex design requires more images, and this translates to a higher circuit depth; increasing the number of images also adds additional qubits to the cycle shift operation (or extended selection). All these constraints slow the performance of the simulation and ask for a different approach to the cycle shift operation (or current image selection). In this section, we focus our attention on the circuit from Figure 13, for which we present its performance in three situations.

For such circuits, the probability histogram is barely visible (which is to be expected, having an increasingly high number of qubits), so the file with the results is the one that we can process after the simulation (the result can be saved in JSON format after the simulation from the code). We decided to inspect two main metrics: simulation time and error; they are obtained using the Qiskit Aer simulator component modelled using the real noise properties of quantum processors (a research path chosen due to the waiting queues required by real devices).

Trying to simulate these circuits on an ordinary desktop or laptop needed a notable amount of time on the CPU, so we approached the tests with the help of a GPU. For the experiments, we installed the python package qiskit-aer-gpu-cu11 (compatible with Nvidia CUDA 11 versions, more info at [36]), and the operating system Ubuntu 22.04.5 LTS. This version choice was motivated by various compatibility issues between the versions of the software packages (all the Cuda toolkit versions for different operating systems and the install instructions can be inspected in the CUDA Toolkit Archive [37]).

The most important details for the configuration of our system are presented in Table 1.

The circuit described in Figure 13 contains the following gates and blocks:

- 12 NOT (X) gates;
- 1 Controlled-NOT (CX) gate;
- 4 Controlled-controlled-NOT (CCX) gates;
- 3 Hadamard gates;
- 2 NEQR blocks.

For our choice of gray values, each NEQR block above requires 15 CCX gates and 8 NOT gates (the Hadamard gates were extracted from the NEQR block in this experiment to be added only once at the beginning of the circuit and have already been counted). So, in total we have:

- 28 NOT (X) gates;
- 1 Controlled-NOT (CX) gate;
- 34 Controlled-controlled-NOT (CCX) gates;
- 3 Hadamard gates.

At the time of writing this paper, the characteristics of the selected quantum devices are presented in Table 2 (the processor types are Heron r2 for IBM Aachen and Eagle r3 for Brussels and Strasbourg). More details on these QPUs can be found at [38] (a free account is required).

### 4.2. Analysis and Discussion

**Figure 14:**
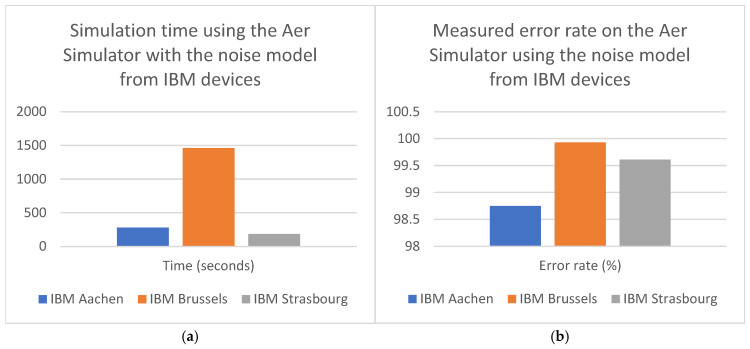
Simulation time (**a**) and measured error charts (**b**) for the experiments ran on the Aer Simulator.

We tested the circuit using the noise model of the devices presented in Table 2; the obtained performance metrics are shown in Table 3, for a number of 4096 shots, and then visually presented in the charts in Figure 14.

In terms of execution time, the performance obtained using the models from IBM Aachen and Strasbourg was relatively similar, clocking around 3–5 min., but for the Brussels model it took almost 25 min.

For the error measurement component, the results obtained are extremely high: over 99% for the noise model of IBM Brussels and Strasbourg, and just below 99% (98.75%) for Aachen. Although close, this confirms the device error hierarchy, with the lowest experimental result corresponding to the lowest error rate from the official website for IBM Aachen (of 6.50 × 10^−3^). This is to be expected; for real processing algorithms used in the industry, quantum companies are slowly beginning to move away from the ideal Simulator due to its limitations (its performance is closely related to the local hardware that it runs on); a perfect environment can be a good starting point, but a real device can provide readers and companies with a more accurate analysis of complex circuits, and lead to further investigation and mitigation of the noise impact, selecting corresponding error correction techniques. However, we should mention a key aspect: our circuit design did not make use of any error correction techniques (as this is not one of the objectives of our paper). This means that (as anticipated) we encountered a very high error rate for all the tested noise models.

In terms of actual methodology for the error measurement, our experiments were conducted using the following approach—we identified, in the output JSON file (with the probability results), the theoretically correct states by taking into account the grayscale values of the pixels and the configured threshold value of 192. For our circuit, the values of the classical register (12 bits) follow the next pattern (as can be seen in Figure 13, expressed from left to right):

- 1 bit for the comparator result (1 if the first two bits of the pixel intensity are 00, 01 or 10, and 0 if they are 11);
- 1 bit for the image selection (0 for the first image, 1 for the second);
- 2 bits for the position (00, 10, 01, 11—as the column index is written first; the column index corresponds to $q_{9}$, and the line index corresponds to $q_{8}$);
- 8 bits for the image representation.

In our case, the 8 values that correspond to what we expect to ideally find after the measurement process are as follows (the last 8 bits are underlined and indicate the gray level):

- 100000000000, 101001001011, 000111001000, 001111111111—for the first image;
- 110000000000, 111001100100, 110110101010, 011111111111—for the second image.

The interpretation of these values is also presented as follows (the comparator result is configured as the first bit of each value and is set in bold):

- For the first image:
  o **1**00000000000: the comparator result is 1; the gray level of the pixel (0) is lower than the threshold value (192);
  o **1**01001001011: the comparator result is 1, 75 < 192;
  o **0**00111001000: the comparator result is 0, 200 > 192;
  o **0**01111111111: the comparator result is 0, 255 > 192;
- For the second image:
  o **1**10000000000: the comparator result is 1, 0 < 192;
  o **1**11001100100: the comparator result is 1, 100 < 192;
  o **1**10110101010: the comparator result is 1, 170 < 192;
  o **0**11111111111: the comparator result is 0, 255 > 192.

From our experiments, the number of counted ideal (expected) states detailed in the previous explanations is as follows (out of 4096 total shots):

- 51 for Aachen (correctly identified percentage: 1.25);
- 3 for Brussels (correctly identified percentage: 0.07);
- 16 for Strasbourg (correctly identified percentage: 0.39).

The full explanation for these results would be the subject of a different, completely experimental paper that measures circuits and various configurations on a range on quantum devices. However, these results could be partially explained by further investigating the exact topology and parameters of the selected quantum processors. The differences are also justified by the circuit mapping, obtained when calling the Qiskit transpile(…) function, which finds the optimal qubit configuration for the selected circuit and quantum processor, given the actual connections—and this comes down to the number of additional gates, with the SWAP gate being one of the most costly in terms of processing time (used to bring together the state of qubits that are not directly connected).

### 4.3. Comparison and Advantages Compared to Other Ideas

The main advantages of our solution, compared to other concepts, are presented in Table 4. It is important to notice that although a significant number of ideas have been proposed over the years in the QIP field, the innovative side of our solution is justified by first combining some of the novel schemes; we then also investigate the performance results obtained for the simulation component using the local GPU, and therefore follow with an experimental analysis of the circuit using the Qiskit software framework.

## 5. Conclusions

This paper highlights a new method that incorporates together different concepts with the purpose of creating a new video processing algorithm using quantum properties. Ideas regarding image representation techniques, cycle shift algorithms (or a selection operation), and compar−ator designs are all put together to build a new way of processing a small sequence of images. The obtained circuit helps us efficiently determine pixel intensities that verify certain properties (in relation to one or multiple thresholds).

The main illustrated circuit can be easily extended; its purpose here was to express a result of a sequence of images in relation to one threshold value. Multiple threshold blocks can be added, thus finding the likely regions that the user is looking for. We can also say that this operation may be used as a prequel to a binarization technique, as the threshold value can be set to the middle value of the desired range; in this manner, we are effectively indicating whether certain pixel values are closer to black or white. This design can also serve as a core component in a chain of image processing layers.

The authors of paper [10] rely on the “reset to $\left|\left.0\right\rangle \right.$“ operation when designing their segmentation circuit (illustrated as the $\left|\left.0\right\rangle \right.$ gate in their circuit). However, in all circumstances we must carefully analyze and consider that this operation, although possible in quantum devices, will collapse the state of the measured qubit, together with the state of any entangled qubits. We should take into account that after applying some CNOT and CCNOT gates—which entangle the auxiliary qubit with color or position qubits—by resetting the auxiliary qubit, we will also affect (and collapse) these entangled qubits. Our design does not involve using such an operation (reset) and is therefore unaffected by this idea. The current frame is indicated by one selection qubit (controlling the NEQR block), set in superposition at the beginning of the circuit, which allows the speedup that gives the quantum solution a clear advantage over a classical one (by processing all the frames in parallel).

The described ideas are tested experimentally, using the local simulator component with the noise models of three quantum devices (IBM Aachen, Brussels and Strasbourg), with the results (time and error rate) presented and analyzed. The experiments were relevant in showing the importance of developing and improving circuits using error correction algorithms. Without them, the tests confirmed high error rates, which would worsen in case of extending the design (by adding more qubits). One research idea that can continue the work presented here is measuring the impact that different error correction methods have on our circuit (in terms of error, processing time or circuit depth); the advancement of both technology and algorithms allows us to generalize such circuits, by using more blocks for the images and perhaps alternative representation techniques.

## Figures and Tables

**Table 1:** The main components of the system used in the experimental analysis.

Testing System Component/ Software Package	Version
Operating system	Ubuntu 22.04.5 LTS
Graphics card (GPU)	Nvidia Geforce RTX 2080 Super
Nvidia driver	550.163.01
Nvidia Cuda toolkit	Cuda compilation tools, V11.7.99
Python Programming Language	3.11.14
qiskit <python package>	2.2.3
qiskit-aer-gpu-cu11 <python package>	0.17.2
qiskit-ibm-runtime <python package>	0.43.1

**Table 2:** A part of the official specifications of the quantum machines made available by IBM.

Device Name (IBM)	ibm_aachen	ibm_brussels	ibm_strasbourg
Qubits	156	127	127
2Q error (layered)	6.50 × 10^−3^	2.36 × 10^−2^	3.50 × 10^−2^

**Table 3:** Performance results for the proposed circuit on different quantum devices.

Noise Model From Device	ibm_aachen	ibm_brussels	ibm_strasbourg
Simulation time (seconds)	278.91	1462.48	184.44
Measured error rate (%)	98.75	99.93	99.61

**Table 4:** Comparison between our idea and related solutions.

Solution	Short Description	Advantages/Drawbacks
An improved two-threshold quantum segmentation algorithm for NEQR image [10]	A segmentation algorithm for images, represented using NEQR, built on multiple threshold comparisons.	$A\;\mathrm{potential}\;\mathrm{problem}\;\mathrm{could}\;\mathrm{be}\;\mathrm{the}\;\mathrm{application}\;\mathrm{of}\;\mathrm{the}\;“\mathrm{reset}\;\mathrm{to}\;\left|\left.0\right\rangle \right.$” operation, which inherently loses the overall quantum advantage (the reset qubit should not be entangled with data qubits).
A quantum moving target segmentation algorithm for grayscale video based on background difference method [11]	An algorithm used to process video sequences, each represented using NEQR, and making use of the background difference method.	The reset operation is still used, with the same potential drawback as previously mentioned.
Our solution	A set of ideas, based on NEQR, that can be used to process a small sequence of images, using a modular design.	We analyze the performance using the simulator and local GPU; the modularity of our design allows us to approach different ideas, according to the configuration of the threshold value. We can replace the image representation method (with similar ideas), test different ways of selecting the current frame, configure (add or remove) the threshold values, and use more image processing ideas at the end of the workflow. We also emphasize (and show) the necessity for error correcting circuits or different designs, as the probability of the desired states drops to extremely low values as the number of used qubits increases.

## Data Availability

Data are contained within the article.
